# With reverence, actions have bounds: the relationship between awe and junior high school students’ self-control—the mediating role of meaning in life

**DOI:** 10.3389/fpsyg.2025.1671015

**Published:** 2026-01-27

**Authors:** Yuhan Zhang

**Affiliations:** Student Affairs Office, Guangxi Minzu Normal Univerisity, Chong Zuo, China

**Keywords:** awe, junior high school students, meaning in life, mediating effect, self-control

## Abstract

**Introduction:**

School bullying is frequent among Chinese junior high school students, and this phenomenon is associated with insufficient reverence and poor self-regulation, making awe education an urgent need. Based on the self-control strength model, prototype model of awe, and hierarchical model of meaning, this study aimed to explore the relationship between awe (including trait and state awe) and self-control among junior high school students, with meaning in life as the mediating variable.

**Methods:**

Two complementary studies were conducted. Study 1 adopted a cross-sectional survey design, administering the Chinese Trait Awe Vocabulary Rating Questionnaire, Self-Control Scale, and Meaning in Life Scale to 476 junior high school students. Study 2 used an experimental design, in which positive and negative state awe were primed among 239 junior high school students to examine causal relationships.

**Results:**

The results revealed two key findings: (1) Meaning in life fully mediated the relationship between trait awe and self-control; (2) Meaning in life partially mediated the relationship between positive-state awe and self-control. Negative-state awe showed no significant mediating pathway with self-control through meaning in life.

**Discussion:**

Consistent with theoretical expectations—where awe enriches meaning in life via schema accommodation and meaning in life enhances self-control by promoting long-term goal pursuit—these findings confirm that awe influences junior high school students’ self-control through the mediating role of meaning in life. This study provides empirical support for the theoretical link between awe and self-regulation, and offers practical insights for designing awe education programs to enhance adolescents’ self-control and reduce school bullying.

## 1 Introduction

In recent years, cases of school bullying and even criminal incidents in junior high schools have become increasingly frequent in China. The underlying cause is that the suspects lack reverence for life and law, as well as the ability to regulate their words and actions, making awe education an urgent priority. Awe plays a crucial role in establishing junior high school students’ outlook on life, values, and the world, as well as their concepts of life, nature, and rules, helping them develop positive and healthy personality traits and excellent virtues (Yu, 2005). Meanwhile, awe can also arouse junior high school students’ cherishing of their own and others’ lives. By understanding that life is a process toward death, they can truly hold awe for life and thereby obtain and understand the meaning of life (Dang and Liu, 2019). Junior high school students are just entering adolescence, a critical period for the formation and development of social emotions. Awe not only provides the endogenous emotional driving force for their personality and social development (Lin et al., 2021) but also promotes their ability to regulate and control their words and deeds (Li, 2017).

### 1.1 Awe

Awe is a complex emotional state that occurs when individuals encounter something grand and beyond their understanding, such as natural wonders, artistic masterpieces, or extraordinary human behaviors, and it is highly contextual (Dong et al., 2013; Pan and Li, 2024; Keltner and Haidt, 2003). According to measurement and induction methods, awe can be divided into state awe and trait awe. State awe is further categorized into positive awe and negative awe based on emotional states (Halstead and Halstead, 2004; Keltner and Haidt, 2003). Positive awe is often accompanied by feelings of peace and joy, and individuals have higher self-control evaluations, while negative awe is associated with fear and powerlessness, characterized by low self-control and high uncertainty (Zhao et al., 2021). Current research mostly focuses on positive awe, with relatively insufficient exploration of negative awe. In addition, in the context of Chinese culture, awe is often regarded as a stable personality trait, and there are significant differences in cognition, emotion, and behavioral tendencies between individuals with and without a “sense of awe” (Zhao et al., 2019). Although studies on trait awe and state awe are mostly separated, the two are not completely disconnected but influence each other closely (Fu and Ling, 2012). Trait awe is the foundation of state awe and acts through states; state awe is the external manifestation of trait awe and can be used to infer individual traits; trait awe can predict short-term state awe, and long-term state awe may also be internalized into stable trait awe (Allen and Potkay, 1981). Based on the above connections, this paper will deeply examine the relationship between awe and self-control of junior high school students from the two dimensions of state awe and trait awe.

### 1.2 The relationship between awe and self-control

Self-control also includes trait self-control and state self-control. At the trait level, self-control is an individual’s ability to disregard or alter internal reactions and interrupt and suppress unwanted behavioral tendencies (Tangney et al., 2004). At the state level, self-control refers to the self’s regulation and control of behavior in specific current situations (Muraven and Baumeister, 2000). The strength theory of self-control mainly proceeds from the state level, arguing that self-control requires the consumption of psychological resources, the total amount of which is limited. Each execution of self-control reduces the total amount of psychological resources, but such resources can also be restored. For example, the induction of positive emotions can increase psychological resources, thereby enhancing self-control (Baumeister et al., 2007; Yu et al., 2016). As a subclass of positive emotions, state awe has been confirmed to enhance people’s self-control performance, such as reducing aggressive behavior (Li et al., 2025; Yang et al., 2016), decreasing the desire for money (Jiang et al., 2018), and weakening the tendency for conspicuous consumption (Xin et al., 2021). In addition, although there is little research on the relationship between trait awe and self-control, Chinese traditional culture has always regarded awe as one of the prerequisites for self-control: “Fear prevents wanton behavior and fosters virtue; lack of fear leads to following one’s desires and courting disaster.” General Secretary Xi has also repeatedly emphasized the importance of “having awe in the heart,” believing that “to stress rules and observe bottom lines, one must first have awe. Only when the heart is in awe can words be restrained and actions be bounded.” Trait awe drives individuals to prudently regulate their words, deeds, and thoughts, and to respectfully deal with matters and interact with others without slackness (Gao, 2021). Therefore, based on the above reasoning, research hypothesis H1 is proposed: trait awe is positively correlated with self-control in junior high school students, and hypothesis H2: state awe can influence the self-control level of junior high school students.

### 1.3 The mediating role of meaning in life

Meaning in life is an adaptive function (King and Hicks, 2021), exerting significant influence on individuals’ physical and mental health (Zhao et al., 2017). Extensive empirical studies have further validated this view: during the COVID-19 pandemic, meaning in life played a critical mediating role in alleviating the negative impact of coronavirus suffering on mental health and life satisfaction (Arslan et al., 2021; Yıldırım and Arslan, 2021; Yıldırım et al., 2020), and it also buffered the adverse effects of loneliness on psychological distress. In non-pandemic contexts, meaning in life has been shown to mediate the relationship between psychological maltreatment and psychological health in young adults (Arslan et al., 2022), as well as the link between occupational stress and psychological distress in teachers (Yildirim et al., 2024).

On the one hand, meaning in life can positively predict self-control, and enhancing self-control is one of its functions (Kesebir and Pyszczynski, 2014). Meaning in life motivates individuals to regulate their behavioral and emotional patterns. This mediating role of meaning in life is not limited to general self-control but also extends to various life domains: for example, it mediates the relationship between school belongingness and adolescents’ internalizing and externalizing problems (Yıldırım et al., 2023), and between resilience and posttraumatic outcomes among earthquake survivors (Türk et al., 2025). According to the hierarchical model of meaning (Schnell and Becker, 2006), meaning is divided into five levels: perception, action, goal, meaning source, and meaning of life. These five levels are not isolated or independent but mutually influential and restrictive. Generally, only when the sense of meaning at lower levels is satisfied can the sense of meaning at higher levels develop. The core of this model is the “goal”—without goals, the sense of meaning cannot exist. To achieve the highest level of meaning—meaning of life—one must restrain immediate, present, and specific desires and instincts, striving for delayed, future-oriented, and grand goals. That is, individuals with higher meaning in life can transcend momentary impulses and instinctive desires to achieve goals, thereby controlling their emotions and behaviors (MacKenzie and Baumeister, 2014).

On the other hand, awe can enhance individuals’ meaning in life. According to the integrated model of meaning making, meaning in life consists of general meaning and situational meaning. When conflict arises between these two types of meaning, an individual’s meaning construction system is activated (Park and Folkman, 1997). Meanwhile, the prototype model of awe posits that “accommodation” is a key element triggering awe experiences (Keltner and Haidt, 2003). Specifically, when individuals encounter unfamiliar or challenging situations that require meaningful adjustment of existing mental schemas, the process of accommodation occurs. During this process, the inconsistency between individuals’ original general meaning and the current situational meaning drives them to actively construct meaning in life, achieving a deeper cognition and understanding of the self and the world. Studies have shown that positive emotions can increase the sense of meaning in life (King et al., 2006). As a positive emotion, awe can positively predict the sense of meaning in life (Dai et al., 2022; Rivera et al., 2020), as can trait awe (Zhao et al., 2018). Trait awe is filled with meaning and a sense of accomplishment (Seaton and Beaumont, 2015), which can broaden horizons, embrace new beliefs (Jiang et al., 2018), increase the possibility of experiencing positive meaning (Fredrickson and Joiner, 2002), and individuals with high trait awe focus on the collective, shifting individual meaning in life toward the group (Shiota et al., 2007), thereby enhancing personal meaning in life. In summary, state awe and trait awe contribute to the discovery and maintenance of meaning in life. Based on the above reasoning, the research hypotheses are proposed: H3: Meaning in life plays a mediating role between junior high school students’ trait awe and self-control; H4: Meaning in life plays a mediating role between junior high school students’ state awe and self-control.

**Figure 1:**
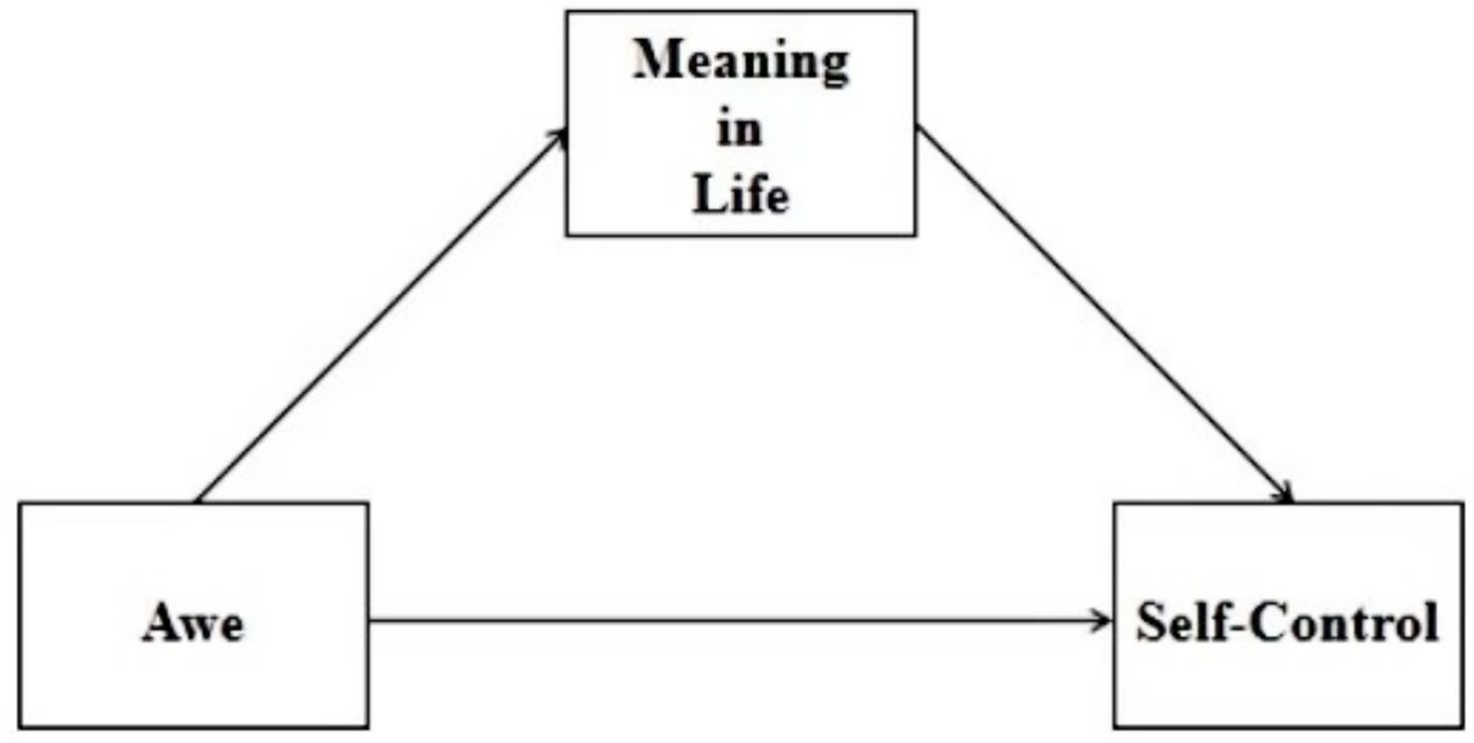
Hypothesized mediating model of awe and self-control in middle school students.

To sum up, this study intends to construct a mediating model as shown in Figure 1. To obtain more robust results, this paper will use the questionnaire method and experimental method to verify the relationship between awe and self-control of junior high school students and the mediating role of meaning in life therein.

## 2 Study 1

### 2.1 Methods

#### 2.1.1 Participants

Using the method of cluster sampling, this study took junior high school students from a certain middle school in Guangxi as the research subjects. Approximately 520 junior high school students were randomly selected from the three grades as participants, and 476 valid questionnaires were recovered, with an effective rate of 91.53%. Among them, there were 207 males (43.49%) and 269 females (56.51%); 187 students in the first grade of junior high school (39.29%), 165 in the second grade (34.66%), and 124 in the third grade (26.05%); 138 only children (28.99%) and 338 non-only children (71.01%).

#### 2.1.2 Research instruments

##### 2.1.2.1 Chinese trait awe vocabulary rating questionnaire

The Chinese Trait Awe Vocabulary Rating Questionnaire developed by Zhao et al. (2019) was used, which better aligns with the understanding of trait awe in Chinese local culture. The scale contains 24 items across 4 dimensions: “Caution,” “Respect,” “Humility,” and “Appreciation.” A representative item is “I am prudent in words and actions.” It uses a 5-point Likert scale (1 = “completely inconsistent,” 5 = “completely consistent”). Except for the item “arrogant,” which is reverse-scored, all other items are forward-scored. Higher scores indicate stronger trait awe. In this study, the scale demonstrates a Cronbach’s alpha coefficient of 0.906, with sub-dimensions showing: Caution (0.866), Respect (0.881), Humility (0.868), and Appreciation (0.829). Confirmatory factor analysis (CFA) confirms good construct validity: *χ*^2^/*df* = 2.300, *RMSEA* = 0.062, *CFI* = 0.926, *TLI* = 0.917.

##### 2.1.2.2 The sense of meaning in life scale

The Sense of Meaning in Life Scale revised by Wang (2013) was adopted, consisting of 10 items with two dimensions: Meaning Possession and Meaning Search. A representative item is “I understand the meaning of my life.” A 7-point Likert scale was used (1 = “completely inconsistent,” 7 = “completely consistent”). Except for the item “My life has no clear purpose,” which is reverse-scored, all other items are forward-scored. Higher scores indicate a stronger sense of meaning in life. In this study, the scale demonstrates a Cronbach’s alpha coefficient of 0.875, with sub-dimensions: Meaning Possession (0.919) and Meaning Search (0.935). Confirmatory factor analysis (CFA) confirms good construct validity: *χ*^2^/*df* = 2.629, *RMSEA* = 0.069, *CFI* = 0.988, *TLI* = 0.985.

##### 2.1.2.3 Chinese version of self-control questionnaire

The Chinese version of the Self-Control Questionnaire revised by Tan and Guo (2008) was used. The questionnaire comprises 19 items with five measurement dimensions: Impulse Control, Healthy Habits, Temptation Resistance, Work Focus, and Entertainment Restraint. A representative item is “I can resist temptation.” A 5-point Likert scale was applied (1 = “completely inconsistent,” 5 = “completely consistent”), where higher scores indicate stronger self-control. In this study, the scale demonstrates a Cronbach’s alpha coefficient of 0.911, with sub-dimensions showing: Impulse Control (0.830), Healthy Habits (0.849), Temptation Resistance (0.771), Work Focus (0.879), and Entertainment Restraint (0.814). Confirmatory factor analysis (CFA) confirms good construct validity: *χ*^2^/*df* = 2.258, *RMSEA* = 0.051, *CFI* = 0.954, *TLI* = 0.945.

#### 2.1.3 Data processing

To ensure data quality, the research team incorporated two attention check items (e.g., “Please select ‘Strongly Agree’ as the answer to this question” and “Please select ‘Strongly Disagree’ as the answer to this question”) to assess whether participants completed the questionnaire carefully. Additionally, we excluded samples with abnormally short response times (less than 25% of the average completion time) or obvious response patterns (e.g., selecting the same option for all items). Based on the aforementioned criteria, a final sample of 476 valid responses was obtained, which were subsequently imported into SPSS and Mplus for data analysis.

### 2.2 Results

#### 2.2.1 Common method bias test

All variables were measured via self-report, potentially introducing common method bias (Podsakoff et al., 2003). To address this, a Harman single-factor test was conducted following the approach recommended by Zhou and Long (2004). Results showed that 11 factors with eigenvalues > 1 were extracted under unrotated conditions, with the first factor explaining 25.71% of variance—below the 40% threshold (Podsakoff et al., 2003). This indicates no severe common method bias in the present study.

#### 2.2.2 Descriptive statistical analysis of main variables

After controlling for three demographic variables (gender, grade, and only-child status), the descriptive statistical analysis of awe, meaning in life, and self-control is shown in Table 1. The results in Table 1 indicate that awe, meaning in life, and self-control among junior high school students are all significantly and positively correlated with each other (*p* < 0.01).

**Table 1:** Descriptive statistics and correlation analysis results (*N* = 476).

Variables	M	SD	1	2	3	4	5	6
1. Gender	-	-	1					
2. Only child status	-	-	0.09*	1				
3. Grade	-	-	0.07	0.16**	1			
4. Trait awe	4.15	0.46	−0.02	−0.11*	−0.11*	1		
5. Meaning in life	5.28	1.06	−0.16**	−0.03	−0.17***	0.50***	1	
6. Self-control	3.57	0.68	−0.14**	−0.03	−0.22***	0.48***	0.50***	1

#### 2.2.3 Mediating effect analysis

With gender, only-child status, and grade as control variables, trait awe, meaning in life, and self-control among junior high school students were designated as the independent variable, mediating variable, and dependent variable, respectively. The mediating effect of meaning in life was tested using PROCESS Model 4 and the bias-corrected percentile Bootstrap method with 5,000 Bootstrap samples and a 95% confidence interval. Results are presented in Table 2.

**Table 2:** Regression analysis of variables.

Regression equation,		Overall fit indices			Significance of regression coefficients	
Outcome variable	Predictor variable	*R*	*R^2^*	*F*	Regression coefficients	*t*
Meaning in life	Gender Only-child status Grade Trait awe	0.54	0.29	49.34	−0.17 0.06 −0.12 0.50	−4.26*** 1.58 −2.97** 12.78***
Self-control	Gender Only-child status Grade Trait awe Meaning in life	0.59	0.35	50.24	−0.10 −0.08 −0.14 0.32 0.30	−2.53* −2.06 −3.70*** 7.37*** 6.77***

The mediating effect is shown in Table 3. The total effect was 0.56, and the direct effect was 0.38. The 95% confidence intervals of both did not include “0,” indicating that both the total effect and direct effect reached statistical significance. This suggests that trait awe among junior high school students is positively related to self-control. The indirect effect was 0.18, and its 95% confidence interval also did not include “0,” confirming the significance of the mediating effect. The mediating effect accounted for approximately 32.14% of the total effect. Therefore, the findings support the research hypothesis: Trait awe among junior high school students is not only positively related to self-control directly but also indirectly positively related to self-control through meaning in life.

**Table 3:** Test analysis of mediating effect significance.

Effect type	Effect	BootSE	BootLLCI	BootULCI
Total effect size	0.56	0.05	0.56	0.65
Direct effect size	0.38	0.05	0.28	0.48
Indirect effect size	0.18	0.03	0.11	0.24

### 2.3 Summary

Study 1 indicates that trait awe among junior high school students is not only directly positively related to self-control but also indirectly positively related to it through meaning in life, verifying Hypotheses H1 and H3. However, since cross-sectional questionnaire results cannot infer causal relationships, Study 2 uses an experimental approach for further verification.

## 3 Study 2

### 3.1 Methods

#### 3.1.1 Participants

Using Gpower 3.1, the required sample size was calculated with an effect size of 0.25, *α* error of 0.05, and power of 0.90, yielding a total of 204 participants. A total of 260 s-grade junior high school students from a middle school in Guangxi were recruited, and after excluding invalid responses, 239 participants were retained, with an effective rate of 91.92%. Invalid questionnaires were excluded based on the following criteria: (1) missing essential information; (2) obvious regular responding; (3) excessive missing items; (4) multiple selections for a single item. There were 104 males (43.51%) and 135 females (56.49%), with a mean age of 13.73 ± 0.52. Participants reported no history of mental illness, had normal or corrected vision, were right-handed, and had not participated in similar experiments before. This study strictly adheres to the Declaration of Helsinki, ethical approval for this study was obtained from the Ethics Committee of the Student Affairs Office of Guangxi Minzu Normal University for research involving human subjects (ethics codes IRB-GXMNU-SAO-2025100502). Informed consent was obtained from all participants, who were subsequently provided with small gifts in acknowledgment of their participation.

#### 3.1.2 Experimental design

A 3 × 2 between-subjects experimental design was used, with the independent variables being awe valence groups (positive awe group, negative awe group, control group) and ego-depletion groups (depletion group, non-depletion group). The dependent variable was self-control (persistence task duration), and the mediating variable was meaning in life (score on the Sense of Meaning in Life Scale).

#### 3.1.3 Experimental materials

1. State awe induction materials. Videos proven effective in inducing specific emotions in previous studies (Rivera et al., 2020; Rudd et al., 2012; van Cappellen and Saroglou, 2012) were used. The positive awe group watched Starry Sky, the negative awe group watched Super Volcano, and the control group watched Chair Making, all approximately 7 min long.
2. State awe induction measurement. Referencing Guan et al. (2019), positive awe emotions (joy, awe), negative awe emotions (fear, awe), and irrelevant emotions (anger, disgust, pride) (Zhao et al., 2021) were used to measure whether state awe was successfully induced. Awe scores were assessed using the Chinese Version of the Awe Experience Scale (Zhou et al., 2022).
3. Depletion task manipulation check materials. Referencing Fan et al. (2019), a 7-point scale was used to evaluate feelings about the depletion task, including three items: “This task was difficult,” “This task consumed a lot of my energy,” and “Completing the task was not easy” (1 = very easy, 7 = very difficult). Higher scores indicated greater ego-depletion.
4. Persistence task. Referencing Fan et al. (2019), two pictures with 12 alleged differences (actual 10 differences) were used.
5. Sense of Meaning in Life Scale. Same as in Study 1.

#### 3.1.4 Experimental tasks

Self-control Resource Depletion Task (hereinafter referred to as the depletion task). This task referred to the Stroop task paradigm (Masicampo and Baumeister, 2011). To enhance the degree of resource depletion, the screen presented four English words (“RED,” “YELLOW,” “BLUE,” “GREEN”) written in four colors. Participants were required to match the color of the words with their literal meanings: press “F” if the word’s meaning matched the font color, and press “J” if they did not. The experiment was divided into 4 groups, with 90 trials per group. In the depletion group, the word meanings consistently mismatched the font colors, while in the non-depletion group, the meanings consistently matched the colors.

Persistence Task. Referencing the study by Fan et al. (2019), participants were shown two complex and similar images with subtle differences. Their task was to find 12 differences (though only 10 existed) within 8 min and mark their positions. Participants could quit midway, and the time they gave up was recorded as a measure of self-control performance. Eligibility criteria required participants to quit after identifying at least 6 differences, ensuring they engaged earnestly in the experiment.

#### 3.1.5 Experimental procedures

Participants were randomly assigned to six groups in a 3 (positive group, negative group, control group) × 2 (depletion group, non-depletion group) design. Upon entering the computer room, they were asked to calm down and begin the experiment in a comfortable, relaxed state. First, participants completed the self-control resource depletion task: the depletion group performed the mismatched word-color task, and the non-depletion group performed the matched task, lasting approximately 7 min. They then filled out a 7-point scale to verify task manipulation. Next, participants watched different video materials for about 7 min, after which they completed the Chinese version of the Awe Experience Scale, the Mood Self-Rating Scale, and the Meaning in Life Scale. Finally, they undertook the persistence task. After the experiment, participants were thanked and given gifts.

### 3.2 Results

#### 3.2.1 Effectiveness tests for resource depletion manipulation, basic emotion, and state awe manipulation

##### 3.2.1.1 Effectiveness test of resource depletion manipulation

Effectiveness of the depletion manipulation was verified by the manipulation check of the depletion task. The depletion group (5.04 ± 0.58) showed significantly higher scores than the non-depletion group (1.62 ± 0.37), *t*(237) = 54.10, *p* < 0.001.

##### 3.2.1.2 Effectiveness test of state awe manipulation

To test the effectiveness of awe emotion priming, one-way ANOVA was used to compare positive awe emotion, negative awe emotion, and irrelevant awe emotion reported by participants in different video priming groups after watching videos (see Table 4). One-way ANOVA and *post hoc* comparisons showed significant differences in positive awe emotion among the three groups [*F*(2, 236) = 1176.43, *p* < 0.001, *η*^2^ = 0.91]. *Post hoc* tests revealed that both the positive group and negative group had significantly higher scores than the control group [*p* < 0.001, 95%*CI* (2.57, 2.80) and *p* < 0.001, 95%*CI* (1.84, 2.06), respectively], and the positive group scored significantly higher than the negative group [*p* < 0.001, 95%*CI* (0.62, 0.85)].

**Table 4:** Effectiveness test of awe emotion priming M(SD).

Emotion type	Positive group (*n* = 76)	Negative group (*n* = 78)	Control group (*n* = 85)	*F*	*η^2^*
Positive awe emotion	3.96 (0.42)^bc^	3.22 (0.41)^ac^	1.27 (0.40)^ab^	1176.73***	0.91
Negative awe emotion	3.14 (0.48)^bc^	3.78 (0.38)^ac^	1.25 (0.16)^ab^	1106.11***	0.90
Irrelevant awe emotion	1.14 (0.19)	1.13 (0.21)	1.15 (0.20)	0.32	0.003

^a^The mean value is significantly different from that of the positive group; ^b^The mean value is significantly different from that of the negative group; ^c^The mean value is significantly different from that of the control group (at the 0.05 level), and the same below.

Significant differences were also found in negative awe emotion among the three groups [*F*(2, 236) = 1106.11, *p* < 0.001, *η*^2^ = 0.90]. Post hoc tests showed that both the negative group and positive group had significantly higher scores than the control group [*p* < 0.001, 95%*CI* (2.43, 2.65) and *p* < 0.001, 95%*CI* (1.78, 2.01), respectively], and the negative group scored significantly higher than the positive group [*p* < 0.001, 95%*CI* (0.53, 0.76)].

No significant differences were found in irrelevant awe emotion among the three groups [*F*(2, 236) = 0.32, *p* > 0.05, *η*^2^ = 0.003]. In summary, the positive group induced more positive awe, the negative group induced more negative awe, both higher than the control group; and there was no significant difference in irrelevant awe emotion among the three groups, indicating that state awe priming with different valences was effective.

#### 3.2.2 Effects of resource depletion manipulation and state awe manipulation on self-control performance

A one-way analysis of variance (ANOVA) was performed on the self-control scores of the three groups of participants (see Table 5). The results were as follows.

**Table 5:** The influence of resource depletion manipulation and state awe manipulation on self-control performance M(SD).

Grouping	Positive group (*n* = 76)	Negative group (*n* = 78)	Control group (*n* = 85)	*F*	*η^2^*
Depletion group (*n* = 122)	475.32 (19.83)	424.73 (61.35)	415.23 (78.72)	11.24***	0.16
Non-depletion group (*n* = 117)	477.56 (11.47)	429.03 (71.16)	424.33 (63.45)	11.20***	0.16
*t*	0.61	0.54	0.59		

Under the resource depletion condition: The main effect of different awe state manipulation groups was significant, *F*(2, 119) = 11.24, *p* < 0.001, *η*^2^ = 0.16. Post-hoc comparisons revealed that the self-control scores of the positive group were significantly higher than those of the negative group and the control group, with values of [*p* < 0.001, 95%*CI* (23.79, 77.38)] and [*p* < 0.001, 95%*CI* (33.44, 86.74)], respectively. Moreover, there was no significant difference in self-control scores between the negative group and the control group [*p* = 0.47, 95%*CI* (−16.28, 35.29)].

Under the non-resource depletion condition: The main effect of different awe state manipulation groups was also significant, *F*(2, 114) = 11.20, *p* < 0.001, *η*^2^ = 0.16. Post-hoc analyses showed that the positive group exhibited significantly higher self-control scores than the negative group and the control group, with [*p* < 0.001, 95%*CI* (23.26, 73.81)] and [*p* < 0.001, 95%*CI* (28.92, 77.55)], respectively. Additionally, no significant difference was observed in self-control scores between the negative group and the control group [*p* = 0.71, 95%*CI* (−20.14, 29.53)].

#### 3.2.3 The influence of resource depletion manipulation and state awe manipulation on the meaning of life

A one-way analysis of variance (ANOVA) was conducted to examine the sense of meaning in life across three groups of participants (see Table 6). The results were as follows:

**Table 6:** The influence of resource depletion manipulation and state awe manipulation on meaning in life M(SD).

Grouping	Positive group (*n* = 76)	Negative group (*n* = 78)	Control group (*n* = 85)	*F*	*η^2^*
Depletion group (*n* = 122)	5.09 (0.96)	5.30 (0.95)	4.59 (1.09)	5.67***	0.09
Non-depletion group (*n* = 117)	5.37 (0.83)	4.96 (0.86)	4.85 (1.25)	2.90	0.05
*t*	1.34	1.67	1.04		

Under the resource depletion condition: A significant main effect of the manipulated awe states was found, *F*(2, 119) = 5.67, *p* = 0.004, *η*^2^ = 0.09. *Post-hoc* comparisons revealed that both the positive and negative awe groups reported significantly higher levels of meaning in life compared to the control group. Specifically, the positive awe group showed a significant difference [*p* = 0.027, 95%*CI* (0.58, 9.49)], as did the negative awe group [*p* = 0.001, 95%*CI* (2.83, 11.45)]. However, no significant difference was observed between the positive and negative awe groups in terms of meaning in life scores [*p* = 0.35, 95%*CI* (−6.89, 2.38)].

Under the non-resource depletion condition: The main effect of the manipulated awe states approached significance, *F*(2, 114) = 2.90, *p* = 0.059, *η*^2^ = 0.05. Post-hoc analyses indicated that the positive awe group reported significantly higher levels of meaning in life than the control group [*p* = 0.023, 95%*CI* (0.73, 9.61)]. There was no significant difference in meaning in life scores between the positive and negative awe groups [*p* = 0.08, 95%*CI* (−0.54, 8.70)], nor between the negative awe group and the control group [*p* = 0.64, 95%*CI* (−3.45, 5.63)].

#### 3.2.4 Test of the mediating effect of meaning in life

To investigate the mediating effects involving multi-category independent variables, this study employed the Bootstrap method developed by Hayes and Preacher (2014) and conducted analyses using the PROCESS 3.3 plugin in SPSS 26.0. Consistent with the plugin’s default coding protocol for dummy variables, the first experimental group was designated as the reference category, and k experimental groups were recoded into k − 1 dummy variables. Specifically, the negative awe group was automatically encoded as dummy variable D1, while the positive awe group was assigned as dummy variable D2.

Under the resource depletion condition, with the control group serving as the baseline, the total effect of positive awe on self-control was estimated at 58.31, with a 95% confidence interval (*CI*) that did not encompass zero, thereby confirming statistical significance. The mediational pathway through meaning in life yielded an effect size of 15.10, and its 95%*CI* also excluded zero, indicating a significant mediating role. Moreover, the direct effect of positive awe on self-control was 43.21, with a 95%*CI* that similarly did not include zero, further validating its significance. In contrast, the total effect of negative awe on self-control was 10.18, and the 95%*CI* of [−15.38, 35.74] spanned zero, suggesting a non-significant relationship.

**Figure 2:**
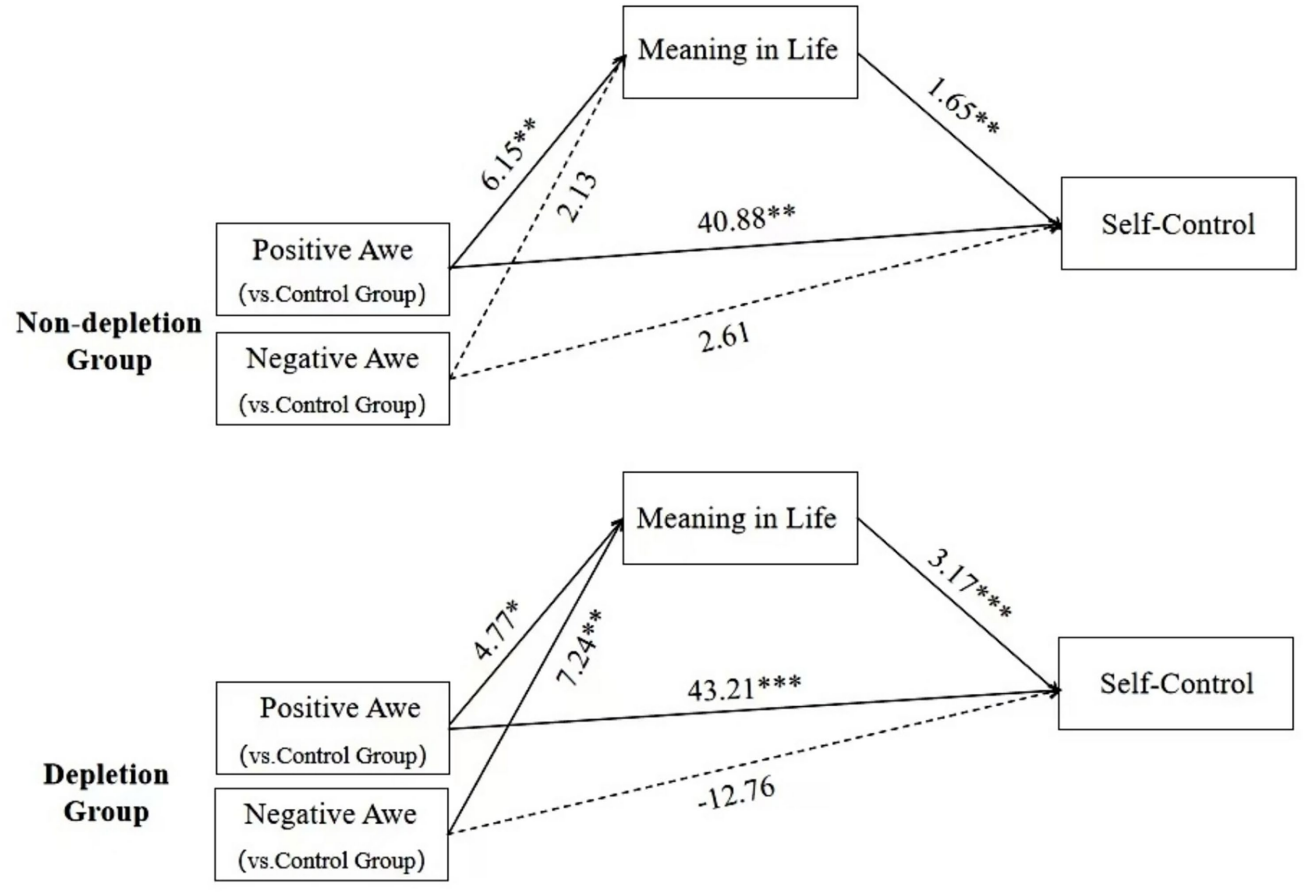
The impact of state awe priming on self-control under different resource depletion managements and the mediating role of meaning in life (The coefficients in the figure are unstandardized coefficients).

Under the non-resource depletion condition, relative to the control group, positive awe demonstrated a total effect of 51.05 on self-control, with a 95%*CI* that did not contain zero, signifying significance. The mediating effect mediated by meaning in life was quantified at 10.17, and its 95%*CI* also excluded zero, indicating a significant mediating mechanism. The direct effect of positive awe on self-control was 40.88, with a 95%*CI* that did not include zero, confirming its statistical significance. Conversely, the total effect of negative awe on self-control was 6.12, and the 95%*CI* of [−19.35, 31.59] included zero, indicating that the relationship was not statistically significant. The results of the analysis for each variable are shown in Table 7 and Figure 2.

**Table 7:** Results of mediating effect analysis of state awe priming on self-control under different resource depletion conditions.

Mediating effect pathways Total effect pathways	Estimate	95% Bootstrap confidence interval	
		BootLLCI	BootULCI
(Using the control group as the reference)			
(Depletion group) Positive awe→Meaning in life→Self-control	15.10	1.20	32.39
(Depletion group) Positive awe→Self-control	43.21	20.45	66.17
(Depletion group) Negative Awe →Meaning in life→Self-control	22.94	9.05	39.64
(Depletion group) Negative awe→Self-control	−12.76	−35.52	10.00
(Non-depletion group) Positive Awe→Meaning in life→Self-control	10.17	1.34	22.78
(Non-depletion group) Positive awe→Self-control	40.88	16.28	65.47
(Non-depletion group) Negative awe→Meaning in life→Self-control	3.52	−4.8	12.46
(Non-depletion group) Negative awe→Self-control	2.61	−21.88	27.10

### 3.3 Summary

The experimental results of Study 2 show that positive awe can enhance self-control among junior high school students both under resource depletion and non-resource depletion conditions. Meaning in life plays a mediating role in both scenarios, indicating that positive state awe not only directly improves junior high school students’ self-control but also indirectly enhances it by increasing their sense of meaning in life. However, negative state awe has no significant effect on the self-control behaviors of junior high school students under either condition.

## 4 General discussion

This study explored the relationship between awe and self-control, as well as the mediating role of meaning in life, which was generally consistent with the hypotheses: trait awe was significantly and positively correlated with self-control, and meaning in life played a partial mediating role in this relationship. Inducing positive state awe not only directly enhanced self-control among junior high school students but also indirectly increased self-control by promoting meaning in life. However, inducing negative state awe did not significantly improve their self-control.

### 4.1 Relationship between awe and self-control

In this study, both trait awe and positive state awe were found to have a significant positive correlation with self-control in junior high school students. Few empirical studies have explored the relationship between trait awe and self-control. Thus, this study inferred their association by inducing state awe and examining its effects. In the experiment, positive state awe was evoked through scenarios of vast starry skies, which stimulated feelings of compliance and appreciation, thereby enhancing self-control. According to Fredrickson (2001) Broaden-and-Build Theory of Positive Emotions, positive emotions can construct individuals’ physical, mental, cognitive, and interpersonal resources. As a positive emotion, awe not only promotes physical and mental health but also mobilizes psychological resources. The adage “With awe in mind, one’s actions have boundaries” reflects that awe experiences can increase self-discipline and self-monitoring (Han et al., 2017). When individuals experience awe toward sacred things, they tend to constrain their behaviors (Wang, 2009). Existing research has shown that state awe can inhibit aggressive behaviors and increase prosocial behaviors (Piff et al., 2015; Yang et al., 2016), suggesting that inducing positive state awe can enhance self-control in junior high school students.

Furthermore, this study adopted Mischel and Shoda's (1995) Cognitive-Affective Personality System (CAPS) model as the theoretical framework to explore the relationship between trait awe and self-control. The CAPS model posits that personality is a dynamic interactive system composed of cognitive-affective units, and although individual behaviors show variability in specific contexts, they maintain certain stability (Shoda et al., 1994). In this study, comparing self-control among the control group and awe-inducing contexts groups revealed that the control group and negative group exhibited higher behavioral variability, while the positive group not only showed reduced behavioral variability but also had significantly higher self-control levels than the other two groups. This finding confirms the dynamic manifestation of trait awe in specific contexts, indicating that inducing state awe can activate individuals’ latent trait awe tendency, enabling them to stably exhibit prosocial behaviors in specific situations. It further reveals the internal connection and transformation mechanism between trait awe and positive state awe, demonstrating that trait awe can positively predict self-control.

However, negative state awe did not significantly enhance self-control in junior high school students, which may be related to the “fear” and other negative emotions embedded in negative state awe. Negative emotions deplete self-control by consuming more cognitive resources—when in negative emotional states, individuals exhibit increased impulsive behaviors such as aggression, overspending, binge eating, and substance abuse (Heatherton and Wagner, 2011; Shachar et al., 2016; Yu et al., 2016). Thus, the impact of negative state awe on self-control involves a dual mechanism: on the one hand, the positive attributes of awe itself may potentially enhance self-control, while on the other hand, the negative emotional components (e.g., fear) weaken self-control. These two effects neutralize each other, ultimately leading to the non-significant impact of negative state awe on self-control observed in this study.

### 4.2 Mediating role of meaning in life

Studies 1 and 2 explored the mediating effects of meaning in life on the relationship between junior high school students’ trait awe, state awe, and self-control. Since the causal relationship between negative state awe and self-control was not significant in this study, we focus on the mediating role of meaning in life between trait awe, positive state awe, and self-control.

First, this study found a significant positive correlation between junior high school students’ trait awe and meaning in life. Previous research has not directly verified this relationship, but indirect evidence supports our findings: Personality serves as the basis for meaning in life experiences (Burton et al., 2015), with agreeableness and openness both significantly positively correlated with meaning in life (Henningsgaard and Arnau, 2008; Lavigne et al., 2013). Trait awe encompasses attributes like “prudence,” “respect,” “humility,” and “appreciation,” which highly overlap with agreeableness and openness in personality (Zhao et al., 2017), explaining the significant positive correlation between trait awe and meaning in life.

Second, we found a significant positive correlation between junior high school students’ meaning in life and self-control ability. Enhancing self-control is one function of meaning in life (Kesebir and Pyszczynski, 2014), as meaning in life motivates individuals to regulate their behavioral and emotional patterns. Individuals lacking meaning in life exhibit low self-control, acting on impulse and instinct. Hedlund (1977) noted that meaning in life is the foundation of personal existence; those with a strong sense of meaning perceive their lives as valuable, know their life direction, and regulate their behavior to align with long-term goals, thus demonstrating stronger self-control. These findings validate Hypothesis 3, confirming that meaning in life mediates the relationship between trait awe and self-control in junior high school students.

Finally, the study found that inducing positive state awe in junior high school students increases their meaning in life, thereby improving self-control. Contrary to Hypothesis H4, meaning in life only mediated the relationship between positive state awe and self-control. Since negative state awe did not significantly impact self-control, its mediating effect was not discussed. Fundamentally, meaning in life is a subjective experience (Heintzelman et al., 2013), intricately linked to emotional experiences. Positive emotions generally correlate positively with meaning in life—individuals with higher positive emotions tend to experience greater meaning in life. Hicks et al. (2012) primed emotions and found that positive emotions significantly enhanced meaning in life. As a subclass of positive emotions, positive state awe similarly increased meaning in life among junior high school students in this study. Concurrently, higher meaning in life was associated with improved self-control, consistent with prior findings of positive correlations between meaning in life and self-control in adolescent and adult samples (Li et al., 2019). Meaning in life is thought to facilitate self-regulation (Damon et al., 2003), providing a basis for long-term goals (MacKenzie and Baumeister, 2014). Guided by meaning, individuals override immediate impulses to control emotions and behaviors in pursuit of goals. In this study, positive state awe induced by awe-themed videos prompted participants to reflect on life meaning, reducing focus on task difficulty and motivating persistence. Notably, the positive group showed a ceiling effect: most participants persisted for the full 8 min, suggesting they might have continued beyond 10 or 15 min.

This study reveals distinct mechanisms through which awe influences self-control in junior high school students, with meaning in life serving as a mediator, offering new insights into their relationship. In educational practice, teachers can cultivate students’ self-control by enhancing positive awe experiences, guiding reflections on life meaning, and reducing problematic behaviors.。.

### 4.3 Theoretical contributes

#### 4.3.1 Theoretical implications for the broaden-and-build theory of positive emotions

This study enriches the practical application of awe in the broaden-and-build theory, suggesting that negative valence awe may not align with the theoretical framework of positive emotions. Although awe is categorized as a subcategory of positive emotions, it comprises both positive and negative valences. Most previous studies have focused on its positive valence, with limited exploration of negative valence. Our findings indicate that negative valence awe does not necessarily fulfill the functional roles of positive emotions.

Firstly, due to the inherent “fear” attribute of negative valence, it failed to enhance participants’ self-control levels as positive awe did in Study 2. Secondly, while negative valence awe can also increase meaning in life, this challenges the assumption that all negative emotional states deplete meaning in life. For example, van Tongeren et al. (2013) primed religious beliefs or concepts of “heaven” and found that individuals in negative emotional states experienced higher meaning in life. Thus, when discussing state awe—especially when applying the broaden-and-build theory—it is crucial to distinguish between positive and negative valences.

#### 4.3.2 Complex influence of negative valence awe on self-control

Previous studies (Heatherton and Wagner, 2011; Shachar et al., 2016; Yu et al., 2016) have suggested that negative emotions generally reduce self-control. However, the impact of negative valence awe is more complex due to its hybrid nature: it is not a pure negative emotion but combines attributes of state awe and fear. This duality means it can evoke both feelings of grandeur and smallness, prompting self-transcendence that may enhance self-control, while simultaneously inducing fearful negative feelings that weaken self-control.

Thus, when examining the effect of negative valence awe on self-control, it is necessary to consider the interactive influence of its dual attributes—one promoting self-transcendence and the other triggering fear—rather than focusing solely on one dimension. This complexity highlights the need for nuanced theoretical frameworks in studying emotions with mixed valences.

### 4.4 Limitations and future directions

This study has several inherent limitations. First, the sample primarily consisted of junior high school students, which may limit representativeness. Future research could expand the sample to include diverse age groups and social populations, enhancing the generalizability of findings. Second, the induction of state awe was relatively monolithic, relying solely on passive video viewing. Future studies may explore more diversified and immersive induction methods, such as virtual reality experiences or on-site natural environment exposure, to strengthen ecological validity. Additionally, the measurement of meaning in life could be enriched. Current approaches relied mainly on self-report scales; integrating objective measures (e.g., behavioral observations or physiological indices) would provide a more comprehensive assessment of its role in the awe-self-control relationship.

Longitudinal designs could be adopted to explore the dynamic effects of awe on self-control over time, addressing the cross-sectional nature of this study. Researchers might also investigate moderating factors (e.g., personality traits, cultural backgrounds) that influence how different valences of awe impact self-control. Furthermore, exploring the neural mechanisms underlying awe’s effects—such as using fMRI to examine brain activity during awe induction—could deepen understanding of its biological basis. Finally, applying findings to real-world interventions (e.g., educational programs or mental health therapies) would bridge the gap between theory and practice, enhancing the practical utility of awe research.

## 5 Conclusion

1. Trait awe can positively predict self-control in junior high school students, and meaning in life plays a mediating role in this relationship.
2. Compared with the control group, positive-valence awe can enhance self-control levels among junior high school students, and meaning in life acts as a mediator in this process.
3. Compared with the control group, negative-valence awe has no significant effect on the self-control levels of junior high school students.

## Data Availability

The raw data supporting the conclusions of this article will be made available by the authors, without undue reservation.
